# Arthroscopic Triple-Loaded Soft Anchor Technique in Medial Meniscal Root Repair: A Systematic, Step-by-Step Approach

**DOI:** 10.1016/j.eats.2022.06.025

**Published:** 2022-10-20

**Authors:** Surasak Srimongkolpitak, Bancha Chernchujit, Thongchai Laohathaimongkol

**Affiliations:** a Orthopedic Department, Faculty of Medicine, Queen Savang Vadhana Memorial Hospital, Si Racha, Thailand; b Department of Orthopedics, Faculty of Medicine, Thammasat University, Bangkok, Thailand

## Abstract

The results of meniscal root repair (MRR) have shown that this repair is the best treatment option in the presence of an acute situation with no degenerative changes. The meniscal root could be restored, in addition to the meniscus's hoop stress function, which is a key component in preventing osteoarthritis progression in the future. Several MRR techniques have been developed, and both improved biomechanics and a lower incidence of failure repair are correlated with suture anchor techniques. Suture anchor techniques also have many ways in which they can be adapted until the disadvantages are eliminated, such as the risk of a major neurovascular problem in the posterior compartment and the difficulty of preparing the base of the meniscal footprint because visualization in the medial compartment of the knee is limited. My MRR technique can improve knee function and allow a return to normal activities without the development of osteoarthritis by relying on the concepts of biomechanics and the regeneration process.

Magnetic resonance imaging (MRI) detection of meniscal root tears has shown a 9.1% prevalence of meniscal root tears, requiring an arthroscopic knee operation. In patients undergoing arthroscopic surgery, the prevalence of medial meniscal root tears has been found to range from 10.1% to 21.4%. The prevalence of lateral meniscal root tears has been found to be 2.8% in patients undergoing all-arthroscopic knee procedures and 8% to 9.8% in those undergoing anterior cruciate ligament reconstruction.^1^

The meniscal root functions as a stabilizing anchor for the meniscus attachment. Meniscal root tear situations are directly related to loss of the hoop stress mechanism. As with a total meniscectomy, the cartilage sustains more damage, resulting in a decreased contact area and higher peak contact pressure.^2^ The results of mid- and long-term meniscal root tear studies have shown that if left untreated, this condition will worsen, resulting in osteoarthritis and the need for knee replacement surgery, as well as the occurrence of spontaneous joint osteonecrosis of the knee.^3^^,^^4^

MRI has been considered a gold-standard modality in the diagnosis of meniscal root tears. The five MRI positive findings were as follows: (1) On a coronal T2-weighted fat-suppressed (T2FS) view of the knee, a herniated meniscus is found outside the margin of the proximal tibia, termed “meniscal extrusion,” (2) A missing meniscus is observed near the posterior cruciate ligament (PCL), known as the “ghost sign,” detected on a coronal T2FS view of the knee, (3) On a coronal T2FS view, a gap is found in the meniscal root, termed the “cleft sign.” (4) On an axial T2FS view, a linear signal is found at the fracture site, and (5) bone marrow is frequently found with bone edema and a contusion under the tear.^5^

The main treatment methods for meniscal root procedures include transtibial pullout repair, suture anchor repair, debridement and meniscectomy, meniscal root reconstruction, high tibial osteotomy (HTO), and extrusion reduction. A systematic review and meta-analysis confirmed that meniscal root repair (MMR) yields better clinical outcomes and radiographic results, as well as lower reoperation rates, than partial meniscectomy.^6^ Many techniques for MMR have been published recently. However, the repair procedures are still being discussed in terms of which is the best for MRR, and they are frequently performed in combination with other operations such as HTO, ligament reconstruction, and plate fixation. The transtibial pullout MMR technique and suture anchor fixation are the 2 mainstay techniques for MRR. Each technique has advantages and disadvantages, and at this moment, researchers are investigating biomechanics, functional outcomes, and meniscal root healing success rates.

The indications for MRR are considered as follows: acute or chronic meniscal root tears with intact articular cartilage or an Outerbridge grade less than 2. A tibiofemoral malalignment, joint space narrowing, and ligament injury must be detected before MRR. For patients with joint space narrowing, malalignment greater than 5° or a body mass index greater than 30 kg/m^2^ constitutes a relative contraindication for MRR, resulting in a high rate of failure.^7^

Preoperatively, a 1-leg standing radiograph of the knee is obtained; in this case, there is no tibiofemoral malalignment and the medial joint space is narrowing. MRI of the injured right knee is performed to determine the accuracy of the cleft sign, which represents a totally torn posterior medial meniscal root (Fig 1, Video 1).

**Fig 1:**
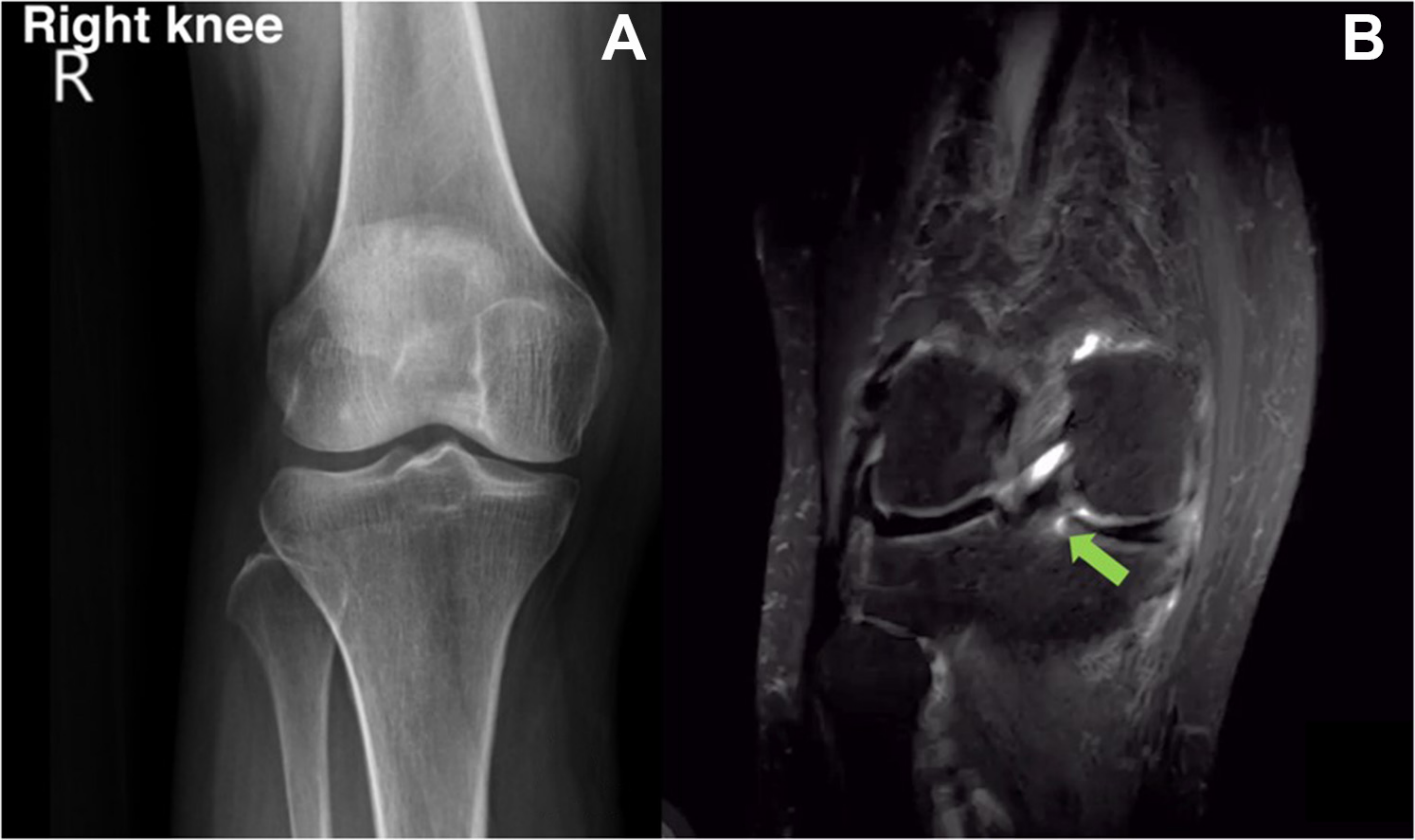
(A) One-leg standing anteroposterior view of the right (R) knee shows no medial joint space narrowing, malalignment of the knee, or osteoarthritis. (B) Magnetic resonance imaging (T2-weighted fat-suppressed coronal view) of the right knee shows that the medial meniscal root is completely torn, as represented by the cleft sign (green arrow).

## Surgical Technique

### Patient Positioning

**Fig 2:**
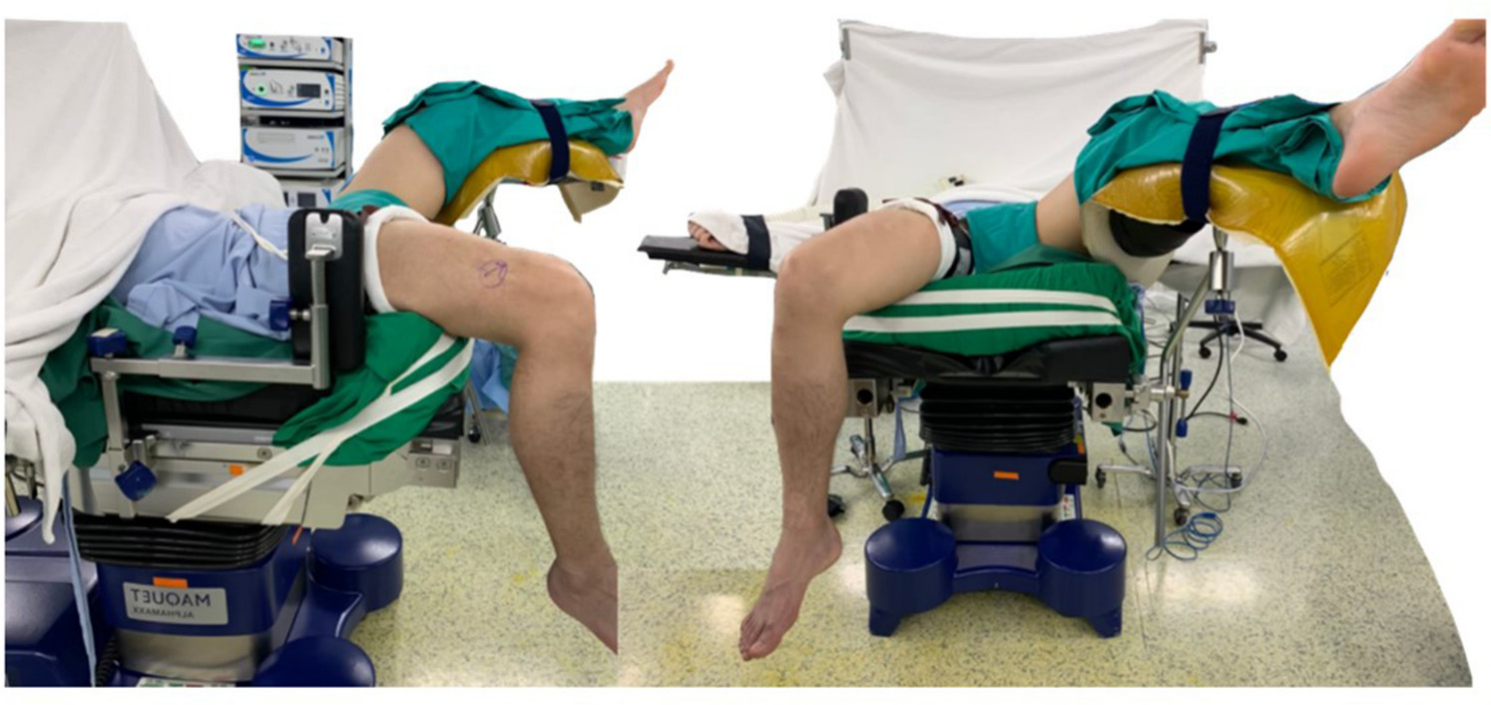
The patient is in the supine position with the affected leg (right leg) hanging and the unaffected leg in a lithotomy position.

The patient is positioned supine on the operating table, with the affected leg in 90° of knee flexion, the posterior thigh secured in a long gel pillow holder, and the affected leg hanging over the side (Fig 2). The unaffected leg is abducted at the hip and placed in a lithotomy holder with sufficient padding. A well-padded pneumatic tourniquet is applied to the proximal thigh. Exsanguination is achieved with an Esmarch bandage before the tourniquet is inflated.

### Portal Placement

Standard anterolateral and anteromedial (AM) parapatellar portals directly adjacent to the patellar tendon are made, together with accessory mid-medial portals as needed. The anterolateral portal, which identifies the posteromedial compartment, is the primary diagnostic viewing portal. The AM and mid-medial accessory portals are the 2 working portals.

### Improved Visualization of Medial Compartment of Knee

**Fig 3:**
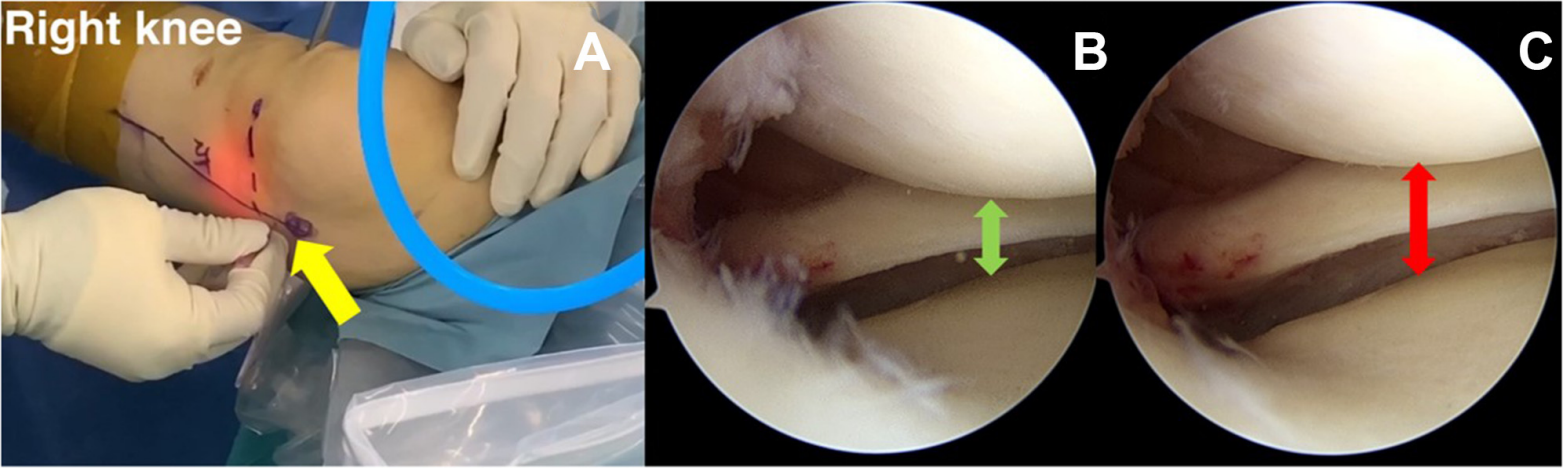
(A) Landmark of posterior medial collateral ligament (MCL) point release (yellow arrow) in right knee. (B) Before MCL release via anterolateral viewing portal (green arrow). (C) After posterior MCL release. Visualization is improved and it is easier to access the posterior medial meniscus via the anterolateral viewing portal (red arrow).

The medial collateral ligament (MCL) causes the medial knee space to narrow the posteromedial knee space and makes it more difficult to place suturing instruments and perform visualization, which is a key problem caused during MRR. Surgical instruments often can cause articular cartilage injury. As a result of this issue, we have proposed a posterior MCL release technique to extend the working space and improve visibility.^8^ An 18-gauge needle is used to perform percutaneous MCL release. The intersection point between the posterior border of the proximal tibial line and the joint line is over 1.2 cm. The knee is flexed to 20° while a valgus force is applied to the tibiofemoral joint and external rotation is applied to the foot. The 18-gauge needle pierces the knee like a pie crust, producing a pop sound and causing the medial compartment to widen. The visualization of the medial working space has greatly improved (Fig 3).

### Creation of Mid-medial Portal

**Fig 4:**
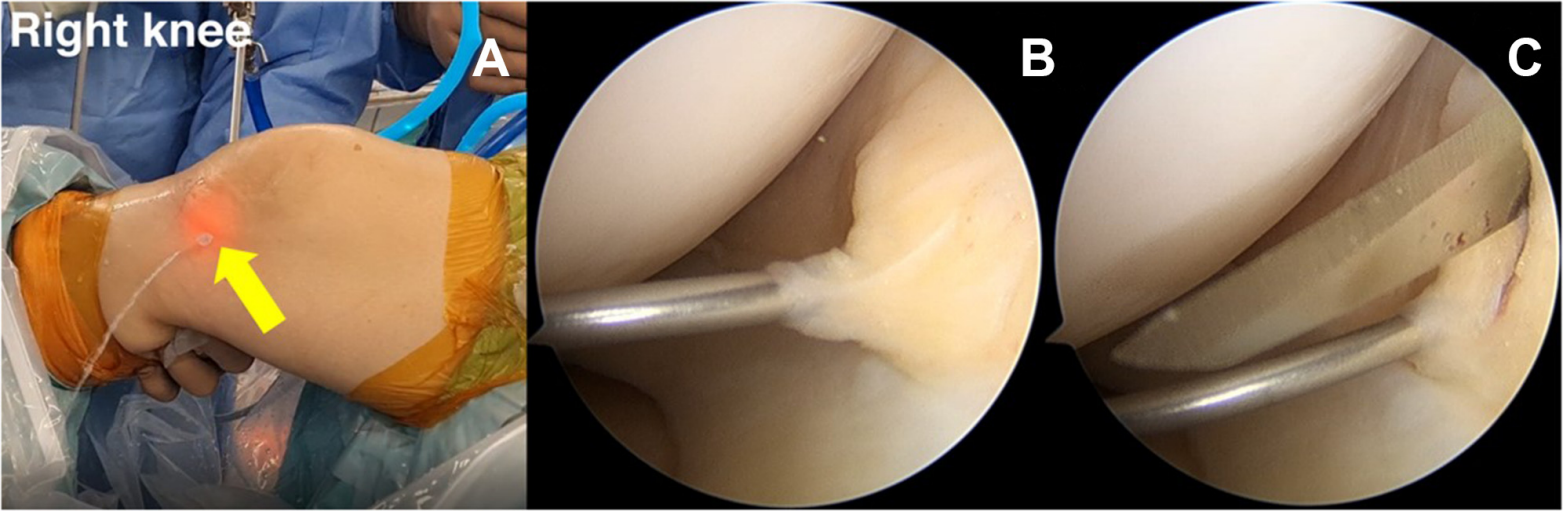
(A) An accessory mid-medial portal is made at the middle part of the medial meniscal body (yellow arrow) in the right knee by use of the arthroscopic light source for a guidance portal. (B) The mid-medial portal creation was guided with an 18-gauge needle by closing the upper part of the medial meniscus body via the anterolateral viewing portal. (C) A vertical stab incision is created by the scalpel blade No. 11. While putting the blade into the knee joint compartment, it should avoid injury to the iatrogenic cartilage and meniscus via the anterolateral viewing portal.

The mid-medial portal is required for posteromedial meniscal root assessment. Because it is close to the posteromedial part of the meniscus, the accessory mid-medial portal has the following advantages: (1) It is easier to insert other instruments. (2) In any surgical procedure, there is no fat tissue that presents a suture management problem. (3) When one is inserting a repair device, injury to the medial femoral condyle cartilage is avoided. The transillumination point from the arthroscopic light source at the upper part of the medial meniscal body is used for the insertion guidance portal. The 18-gauge needle pierces the center of the transillumination point (outside view) and closes the upper part of the medial meniscus body (arthroscopy view). A vertical stab incision is performed parallel to the MCL fiber (Fig 4).

### Meniscal Root Footprint Preparation and Proximal Tibial Tunnel Drilling

**Fig 5:**
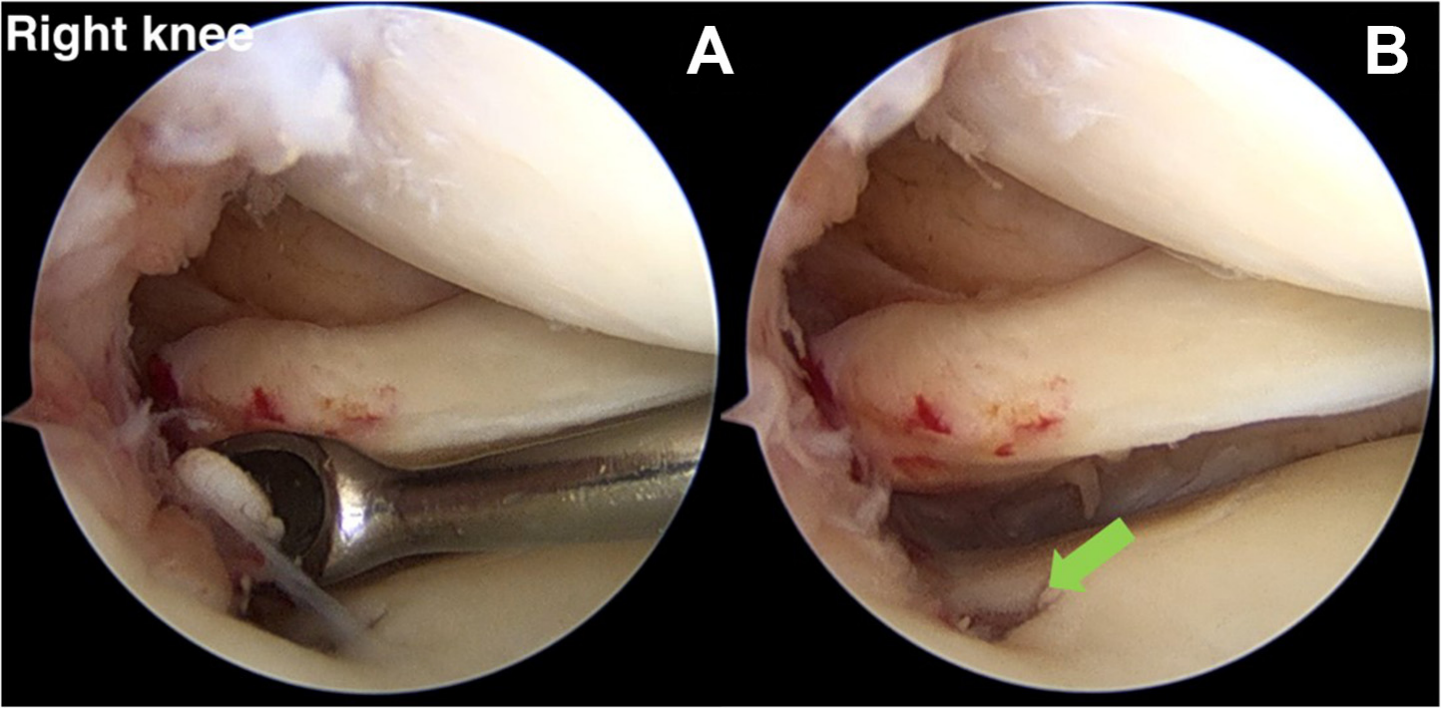
(A) By use of the accessory mid-medial working portal, a round curette is used to remove cartilage until it reaches the subchondral bone (anterolateral viewing portal in right knee). (B) Medial meniscal root footprint preparation at the right tibial site (green arrow; anterolateral viewing portal in the right knee).

**Fig 6:**
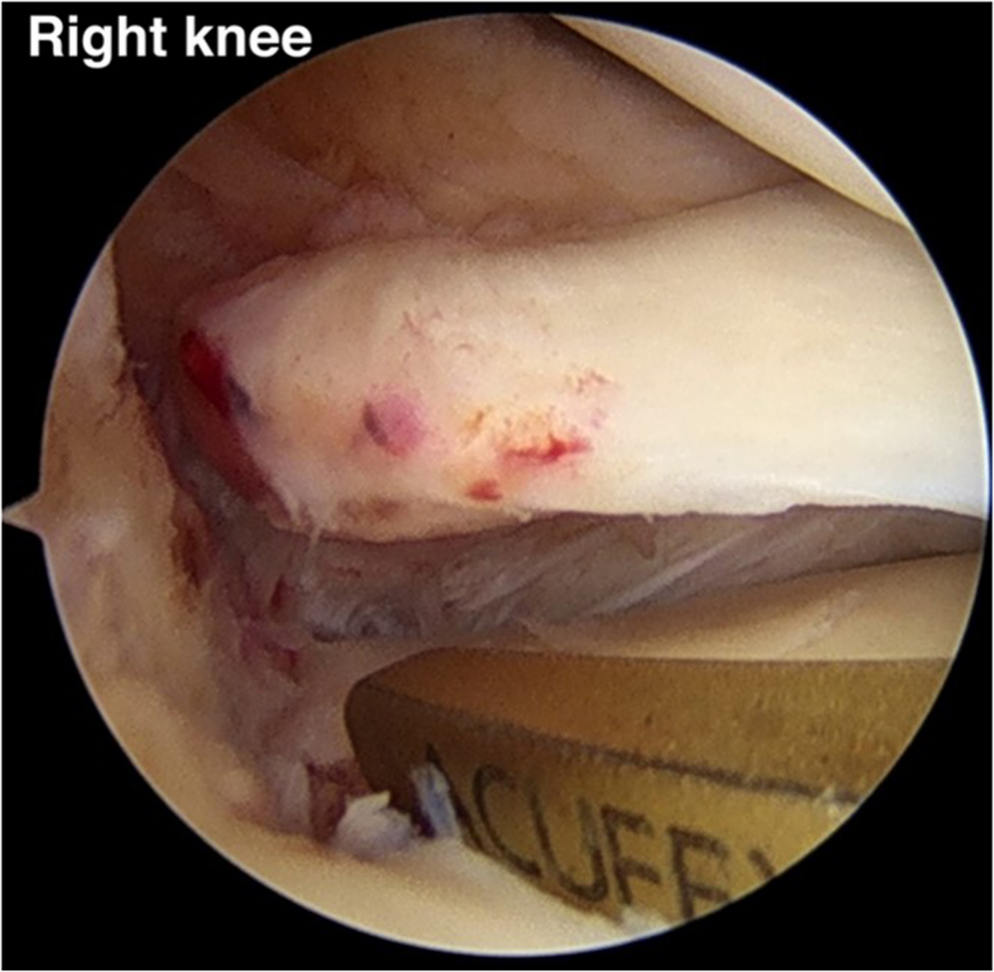
A low-profile aiming device (Acufex Director Drill Guide) is guided to create the proximal tibial tunnel via the anteromedial working portal (anterolateral viewing portal in right knee).

The footprint of the medial meniscal root is debrided with a ring curette via the mid-medial portal, and the cartilage is removed until the subchondral bone is exposed (Fig 5). A flat tip aiming device (Acufex Director Drill Guide; Smith & Nephew) is used for the guidance from the anteromedial proximal tibia to the posteromedial meniscal root footprint. Because of its flat design, this low-profile aiming device is easier to operate and does not injure the medial femoral condyle cartilage (Fig 6). A low-profile aiming meniscal root guide is used to position a drill pin with a cannulated sleeve in the footprint’s aspect, followed by a drill hole with a cannula diameter of about 2.9 mm. The tunnel drilling has the biological consequence of allowing growth factors and progenitor cells from bone marrow to be released, resulting in improved MRR.^9^

### Triple-Loaded Soft Anchor Deployment

**Fig 7:**
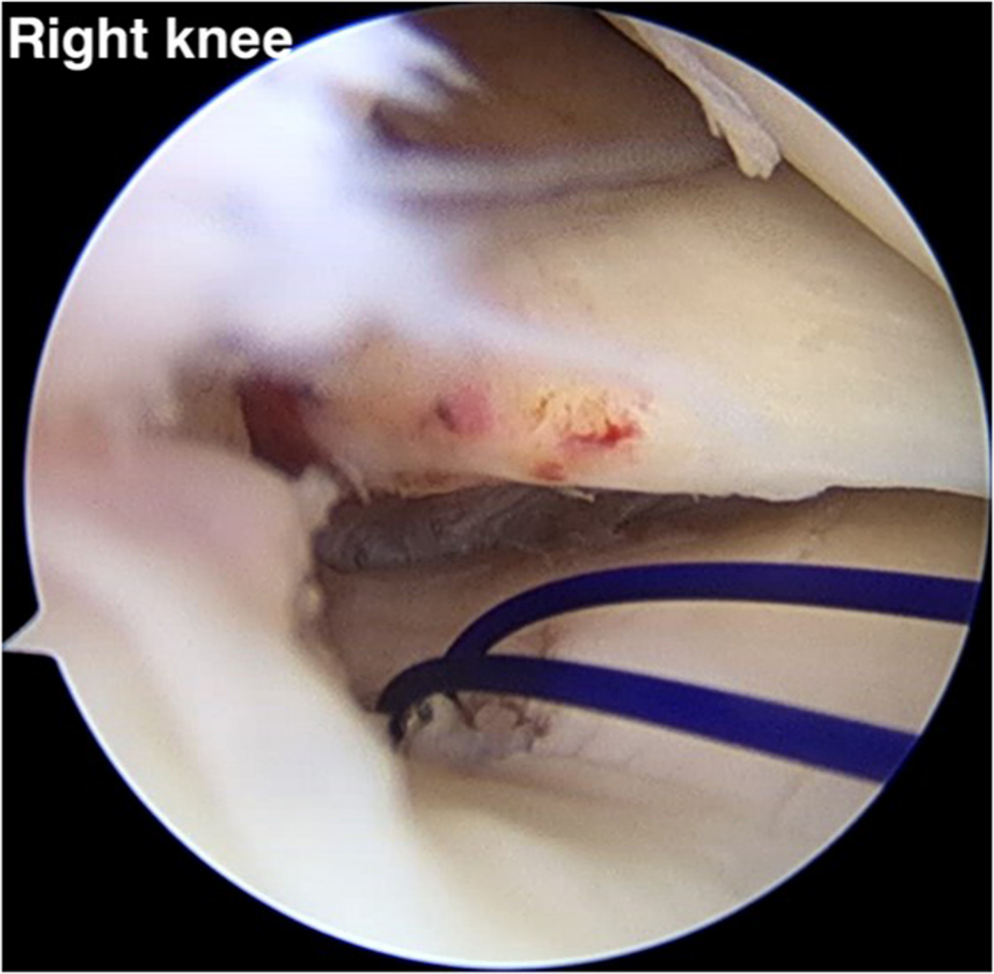
No. 2-0 polydioxanone suture (PDS II) is inserted retrograde into the tibial footprint via a 2.9-mm cannulated drill and is withdrawn to the outside of the anteromedial portal (anterolateral viewing portal in right knee).

**Fig 8:**
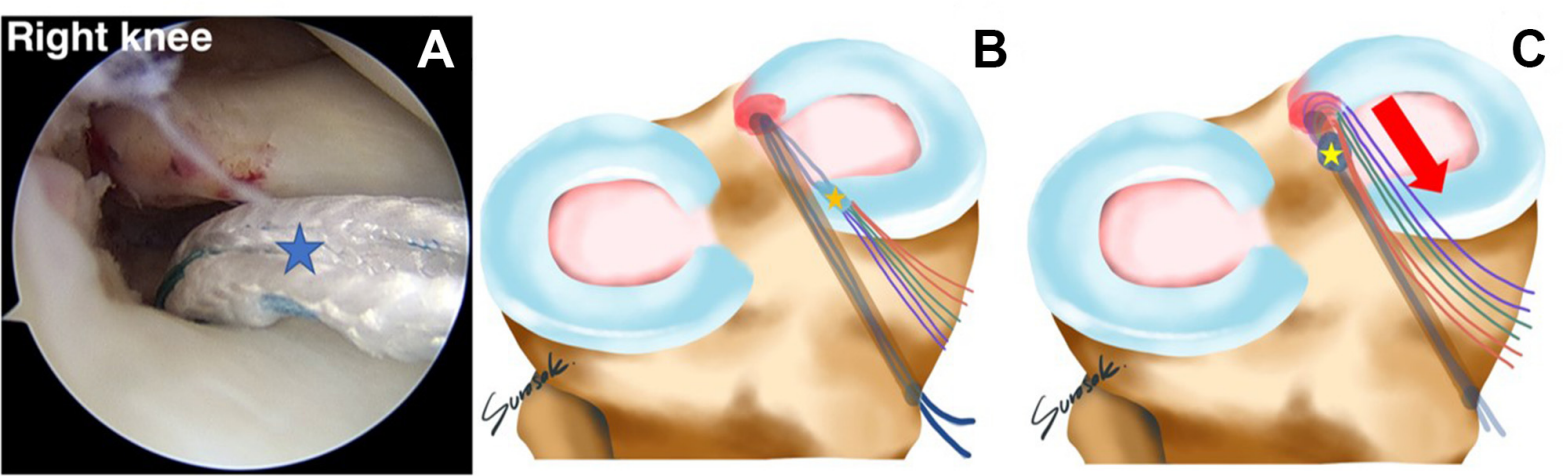
(A) A triple-loaded soft anchor (Y-Knot PRO RC, 2.8 mm) is dragged into the tibial tunnel with a depth of 2 cm (blue star) (anterolateral viewing portal in right knee). (B) Before soft anchor deployment, a spindle shape (orange star) is present. (C) The soft anchor is deployed in the shape of a ball (yellow star) and is securely fixed into the tibial tunnel by pulling the all-suture limb in the direction indicated (red arrow).

A No. 2-0 polydioxanone (PDS) loop (No. 2-0 PDS II suture; Ethicon) is inserted into the cannulated drill at the anteromedial proximal tibia until it appears at the tip of the cannulated drill. A suture retriever is used to retrieve the No. 2-0 PDS (PDS II suture) to the outside via the AM portal (Fig 7). The tip of a triple-loaded soft anchor (Y-Knot PRO RC, 2.8 mm; ConMed) is sutured with Ultratape (Smith & Nephew) to pull the triple-loaded suture anchor into the tibial tunnel. Fiber tape is used to shuttle the loop of the No. 2-0 PDS (PDS II suture) until it reaches the soft anchor suture (Y-Knot PRO RC, 2.8 mm) close to the medial meniscal root footprint. A surgical pen should be used to mark the depth of the soft anchor suture, which is indicated by 1 cm and 2 cm. In our opinion, the 2-cm depth of the soft anchor suture is appropriate for deployment of the soft anchor suture (Y-Knot PRO RC, 2.8 mm) and has no effect on tunnel convergence when performing the other procedures (HTO, ligament reconstruction, and so on). The Ultratape with triple-loaded anchor suture is pulled down through the anteromedial proximal tibia to a depth of 2 cm of the soft anchor suture (Y-Knot PRO RC, 2.8 mm), which changes from a spindle shape to a ball shape with secure fixation into the tibial tunnel (Fig 8).

### Suture Stitch Reduction (Vertical Suture)

**Fig 9:**
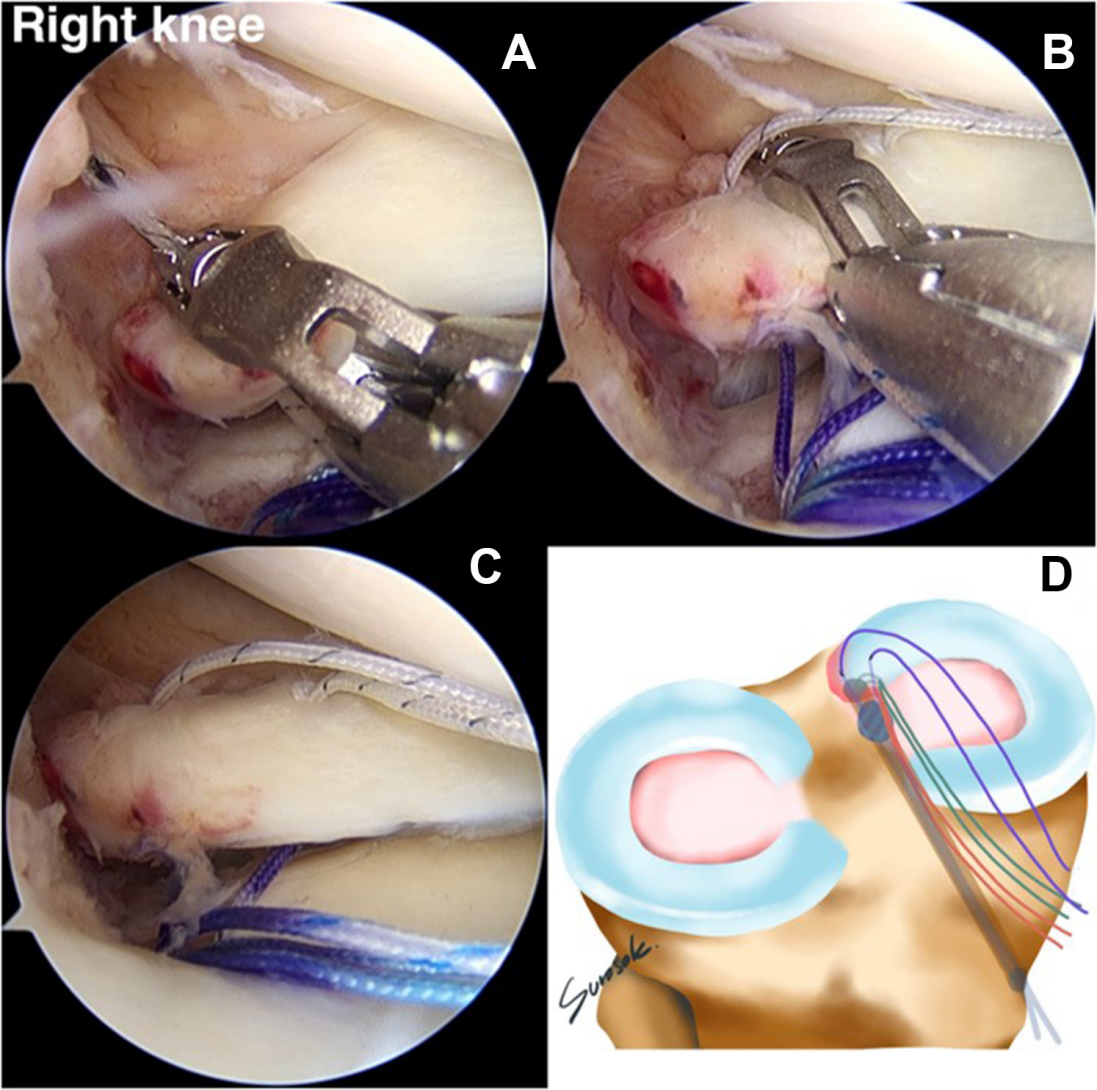
(A, B) A retrograde suture instrument (FirstPass Mini Suture Passer) applies the vertical suture (reduction stitch) to the medial meniscal root via the anteromedial working portal (anterolateral viewing portal in right knee). (C) Completed reduction stitch. (D) The reduction stitch and overall other suture limbs have been placed.

A suture retriever is used to retrieve 1 suture limb to outside the knee via the AM portal and is then loaded with the FirstPass Mini Suture Passer (Smith & Nephew). The FirstPass Mini Suture Passer performs suturing at the 3-mm medial part of the medial meniscal root tear site. The other pair of previously sutured limbs is retrieved from the soft anchor suture and loaded with the FirstPass Mini Suture Passer at the AM portal. The second vertical stitch is sutured far from the first stitch, by approximately 5 mm. The vertical stitch is a reduction stitch that reduces the extruded meniscus and restores the native meniscus (Fig 9). For the next step, the reduction stitch is retrieved to the mid-medial portal.

### Medial and Lateral Compression Suture Stitch (Horizontal Suture)

**Fig 10:**
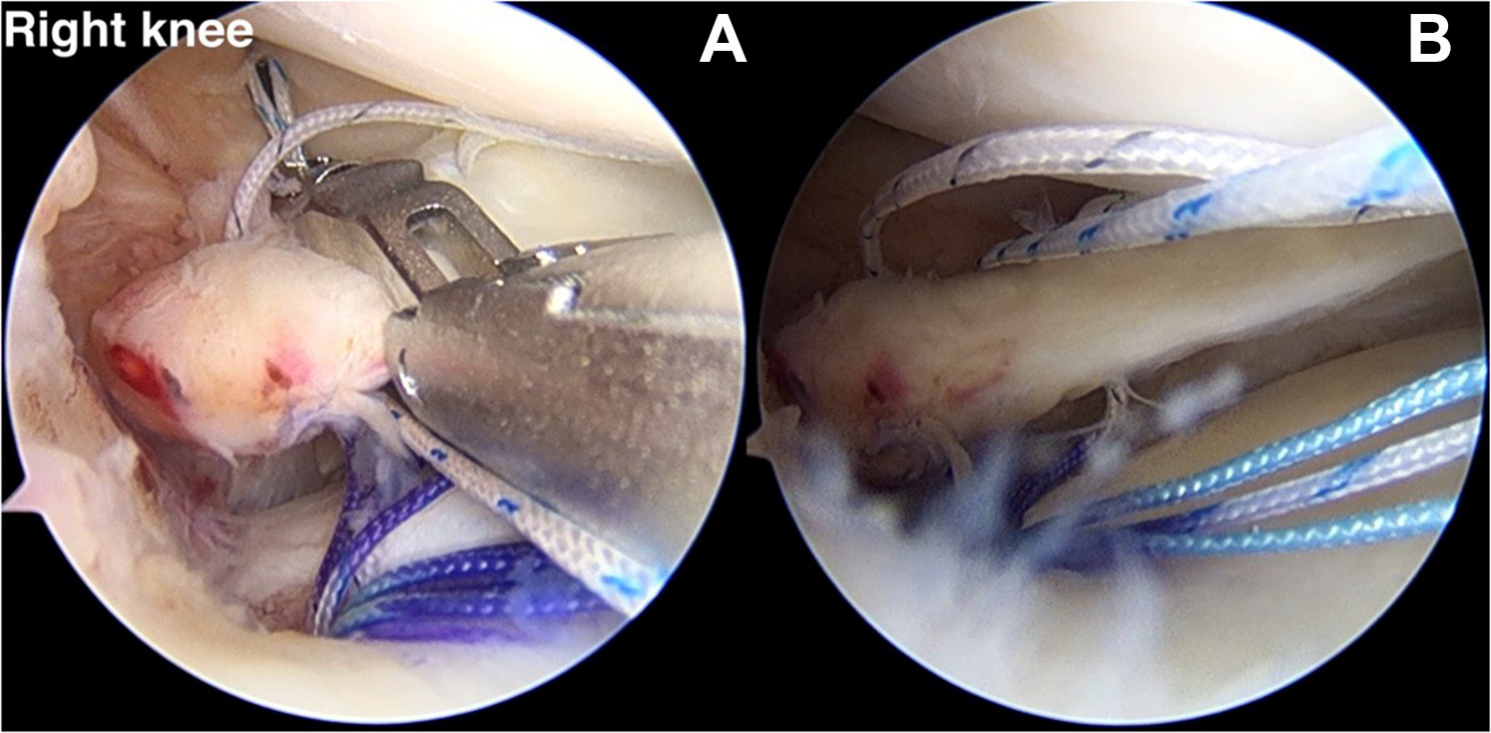
(A, B) The FirstPass Mini Suture Passer performs retrograde suturing of the medial meniscal root with a horizontal suture (medial compression stitch) via the anteromedial working portal (anterolateral viewing portal in right knee).

**Fig 11:**
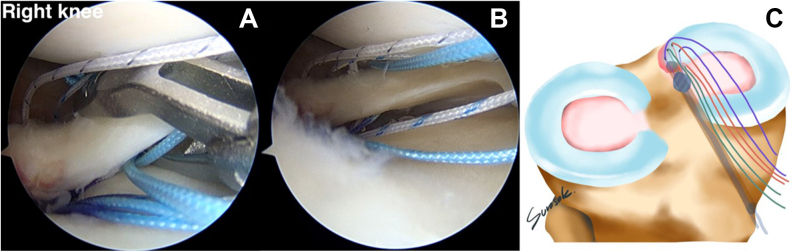
(A, B) The FirstPass Mini Suture Passer performs retrograde suturing of the medial meniscal root with a horizontal suture (lateral compression stitch) via the anteromedial working portal (anterolateral viewing portal in right knee). (C) All the reduction and compression stitches have been completed, in addition to the overall other suture limbs.

By use of the AM portal, the suture retriever is used to retrieve a different pair of suture limbs. The FirstPass Mini Suture Passer is loaded with a looped suture limb, allowing a loop to be passed up through the posteromedial part of the meniscal root and retrieved in a single pass via the AM portal. This stitch will represent the medial compression of the medial meniscal root (Fig 10). The lateral compression stitch is performed repeatedly step by step with the FirstPass Mini Suture Passer. This suture limb undergoes retrograde suturing far from the first horizontal stitch, around 5 mm. The last horizontal stitch represents the lateral compression stitch (Fig 11).

### Tying of Reduction and Compression Stitch Knots

**Fig 12:**
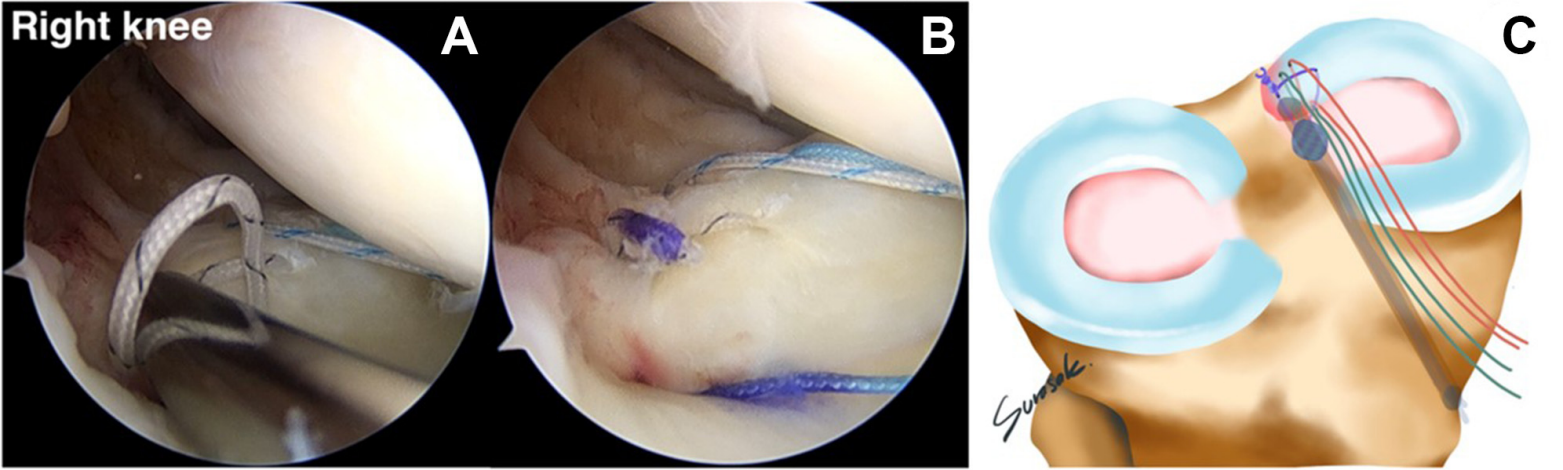
(A) By use of the anteromedial working portal, the reduction stitch knot is tied and the post of the notch pusher is adjusted at the medial part of the reduction stitch (anterolateral viewing portal in right knee). (B) The medial meniscal root is reduced to the native footprint after knot tying. (C) Only the reduction knot has been tied, in addition to the overall other suture limbs.

**Fig 13:**
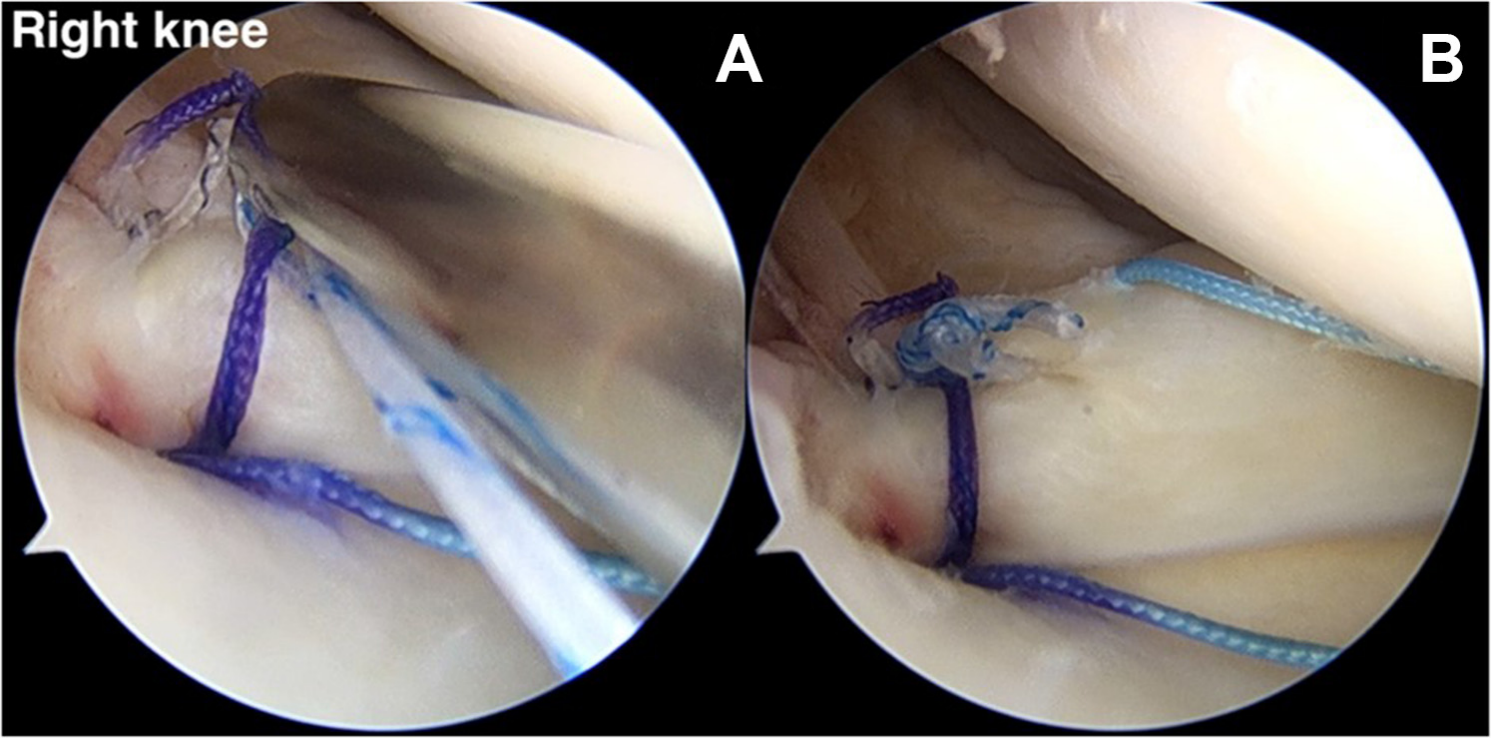
(A) Second knot tying, via anteromedial working portal. The medial compression stitch knot is tied, and the post of the notch pusher is adjusted to the posterior part of the medial meniscal root (anterolateral viewing portal in right knee). (B) The medial part of the medial meniscal root is compressed to the native footprint after knot tying.

**Fig 14:**
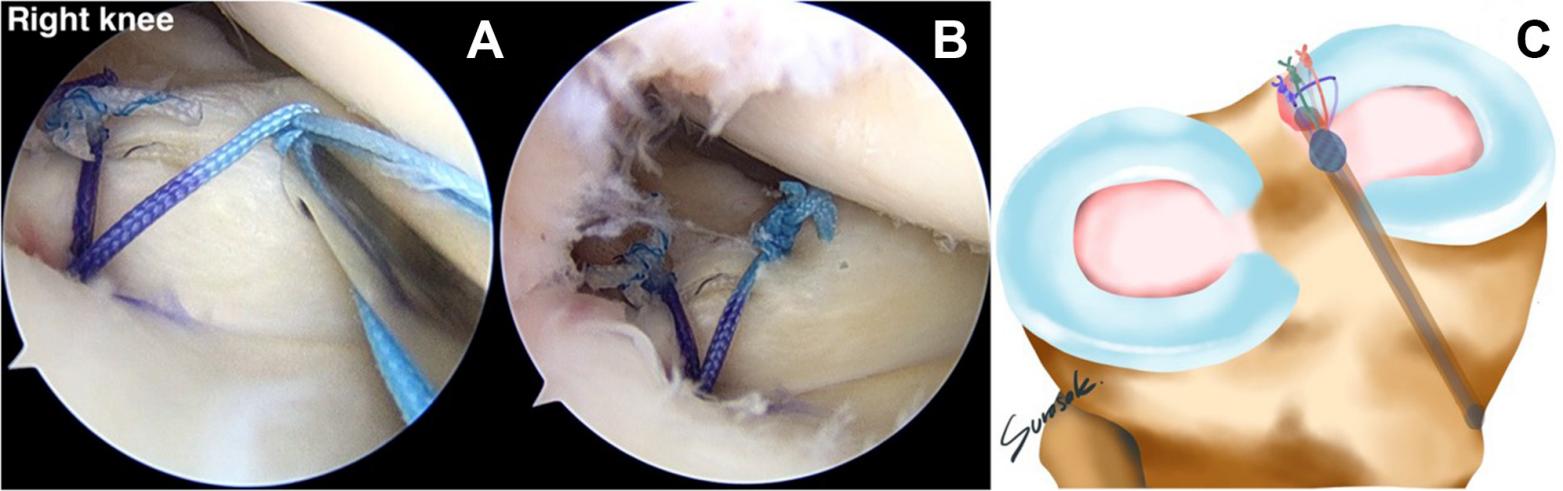
(A) Third knot tying, via anteromedial working portal. The lateral compression stitch knot is tied, and the post of the notch pusher is adjusted to the posterior section of the medial meniscal root (anterolateral viewing portal in right knee). (B) The lateral part of the medial meniscal root is compressed to the native footprint after knot tying. (C) Both reduction knot tying and compression knot tying have been performed.

**Fig 15:**
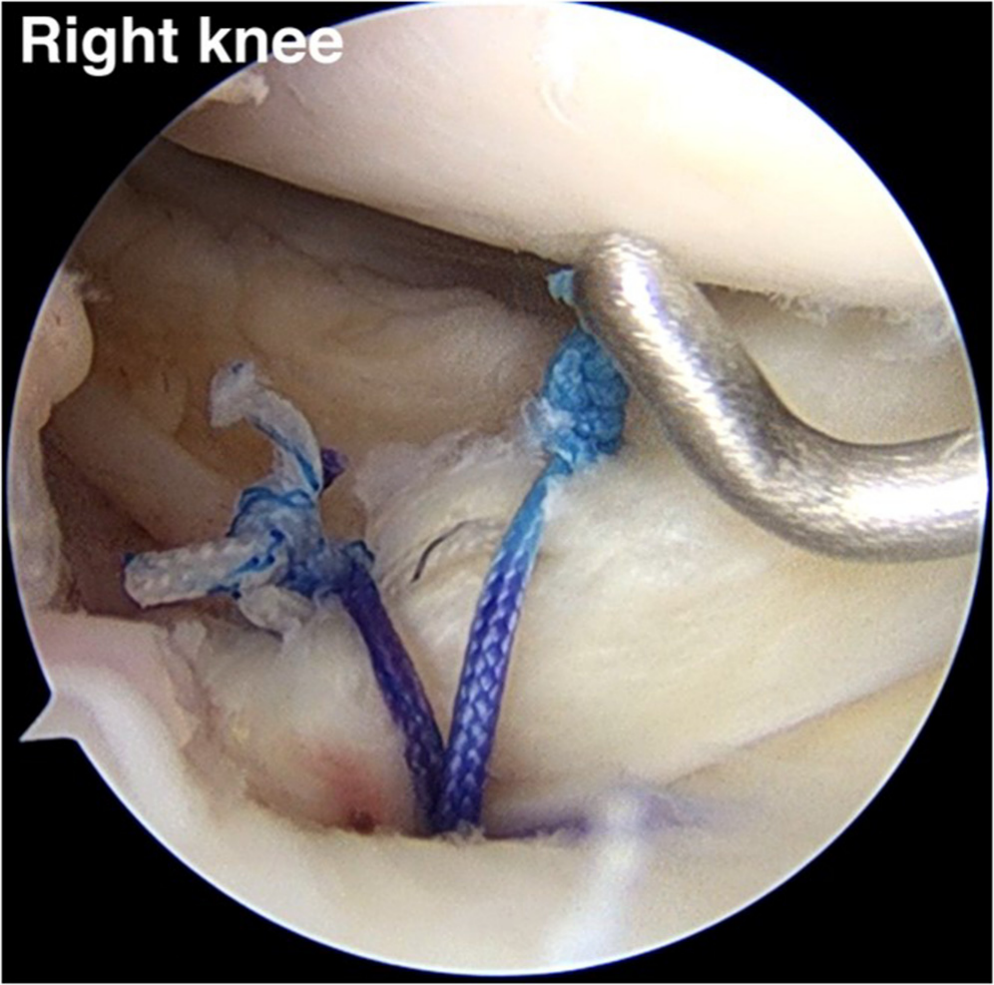
A probe is used to evaluate the stability of the suture configuration and construction via the anteromedial working portal (anterolateral viewing portal in right knee).

The stitches should be knotted in the following sequence: reduction stitch, medial compression stitch, and lateral compression stitch. The first reduction stitch is tied with a knot pusher, with the knot pusher’s post placed at the medial suture limb of the reduction stitch. The knot tying is done one by one, totally four knots. The knots should be tied on the medial side, near the intercondylar notch, to avoid damaging the medial femoral cartilage (Fig 12). The knot in the medial compression stitch is the second knot to tie, and the post of the knot pusher should be in the posterior part of the medial meniscal root. The four-knot is tied one by one via the AM portal (Fig 13). The final knot to tie is the lateral compression stitch knot, which is performed by repeating the previous steps one by one (Fig 14). The suture configuration is comparable to that of a Mason-Allen suturing effect, which results in surface contact rather than suture point contact. The medial meniscal root footprint will be entirely compressed by an all-suture configuration, resulting in a higher potential for healing. A probe is applied to examine the stability of the medial meniscal root restoration site (Fig 15).

### Postoperative Rehabilitation

Patients who undergo MRR are shown to have good clinical outcomes when they receive postoperative rehabilitation. Patients are allowed to bear weight on the tip toes, and the range of motion is limited to 0° to 90° within a hinged knee brace. After 6 weeks, weight bearing can be gradually increased, and 3 months after surgery, patients are able to start normal activities. Knee flexion greater than 90° should be avoided for at least 4 months, and no squatting or jumping should be performed for at least 6 months.

## Discussion

In comparison to traditional suture anchor techniques, the triple-loaded soft suture anchor construct provides significantly lower displacement and increased stiffness, as well as good biomechanical properties. The medial and lateral compression stitches will increase the contact surface healing as a result of enhanced meniscal root healing, thus improving knee function. The tibial tunnel drilling and bed preparation of the meniscal root footprint will enhance the healing rate of the meniscal root; hence, this technique brings the advantages of the transtibial pullout technique while eliminating its disadvantages. Furthermore, extrusion reduction can occur with this method because the reduction stitch will automatically reduce extrusion after knot tying. For these reasons, the triple-loaded soft suture anchor technique is one of the MRR techniques of choice (Tables 1 and 2).

**Table 1:** Pearls and Tips

**Surgical Step**	**Pearls and Tips**	**Pitfalls**
1. MCL release (“magic point” released)	The working room is increased, and it is easier to access the posterior meniscal root site.	Releasing the total part of the MCL should be avoided. Excessive MCL release can lead to loss of valgus stability.
2. Creation of mid-medial portal	The insertion instrument to the posteromedial meniscal root footprint is helpful. Any instrument-related cartilage damage should be avoided. The MCL avoids further injury owing to a vertical stab incision.	To avoid an iatrogenic radial tear of the meniscus, the the needle No. 18 should be guided before the vertical stab incision is created.
3. Meniscal root footprint preparation and proximal tibial tunnel drilling	A low-profile aiming device (Acufex Director Drill Guide) is used to reduce cartilage damage and make it easier to reach the native footprint in which the amount of room is narrowing.	The sharp part of the drill bit that can injure the cartilage should be covered by a round curette. A 2.9-mm-diameter cannulated drill is chosen. If the soft anchor diameter is >3 mm, the soft anchor fixation device (Y-Knot PRO RC, 2.8 mm) will be un**-**deployed.
4. Triple-loaded soft anchor deployment	After soft anchor deployment (Y-Knot PRO RC, 2.8 mm), the secure fixation is tested by pulling the suture limbs. The proper depth of the soft anchor insertion, which should be 2 cm into the tunnel, is indicated with a surgical pen.	The surgeon should not pull the suture limbs too soon after the antegrade soft anchor has been dragged into the tibial tunnel for deployment because the soft anchor (Y-Knot PRO RC, 2.8 mm) will become a ball shape early and will be unable to be implanted and deployed into the tibial tunnel.
5. Reduction and compression suture stitch	The healing surface contact area of the meniscal root is increased.	The accuracy of the retrograde suture implant is critical; if the surgeon performs several penetrations into the meniscus, there is a high likelihood that cut-through will occur when tying the knot.
6. Tying of reduction and compression stitch knots	The reduction stitch (vertical suture) should be tied first, followed by the medial compression stitch (horizontal suture) and, finally, the lateral compression stitch (horizontal suture).	So that the knot will not injure the cartilage and cause irritation, the knot pusher's post should be medial and posterior to the medial meniscal root.

MCL, medial collateral ligament.

**Table 2:** Advantages, Limitations, and Disadvantages

Advantages
There is no bungee effect from the long-length tibial tunnel.
Surface contact area healing is increased.
The soft anchor deployment is a secure fixation.
Tunnel jamming is eliminated if any auxiliary procedure is being performed.
The tibial tunnel drilling enhances biological healing.
The suture anchor construct provides significantly lower displacement and higher stiffness.
Limitations and disadvantages
The suture management procedure should be carefully planned to avoid tangled suture limbs.
The knot-tying placement should be posterior and medial to the meniscus to avoid cartilage abrasion from the knot.
To avoid the soft anchor suture being un-deployed, the cannulated drill should select a diameter that is ≤3 mm.

Anatomically, the native attachment of the posteromedial meniscal root is 8.2 mm in front of the PCL whereas the attachment point of the posterolateral meniscal root is 12.7 mm in front of the PCL.^10^ Anatomic repair is essential for good functional outcomes and a lower repair failure rate. In terms of performing a nonanatomic repair, a previous study found that displacement more than 3 to 5 mm medial to the native site significantly increased the mean contact pressure in the tibiofemoral area until osteoarthritis progressed.^11^

After arthroscopic treatment, partial MCL release has no effect on valgus knee instability. A release of the MCL in tight knees allows for easier visualization of posterior medial meniscal tears and a better comprehension of tear types, minimizing iatrogenic chondral injury.^12^

The transtibial pullout method^3^ is the most widely used method of MMR. This technique is mechanically robust and does not necessitate the use of specialized equipment. The benefits include the potential to enable anatomic restoration, high precision, and a release of blood from bone marrow coming from the tibial tunnel that promotes healing. This technique is still debatable as to whether it causes increased vulnerability to the “bungee” effect. The long-length suture construct and the tibial tunnel length cause micromotion of the meniscal root.^13^^,^^14^ Furthermore, if additional surgical procedures are required, such as HTO or ligament reconstruction, there will be a substantial risk of tunnel convergence or tunnel jamming, in which the suture material will be torn until the construction fixation fails. The advantage of suture anchor repair is that it eliminates the risk of tunnel convergence when auxiliary treatment is performed. The bungee effect, which causes repair failure and micromotion displacement of the repair site, is not present. The anchor placement can be problematic or require opening of the posteromedial portal, which exposes the patient to the risk of vascular and nerve structural injury behind the knee.^15^ Balke et al.^16^ described a soft anchor technique that eliminated the requirement for an incision behind the knee (posteromedial incision). The soft anchor technique could be used in combination with other procedures such as HTO or ligament reconstruction without there being any tunnel convergence issues.

Kim et al.^17^ compared the gap reduction after MMR between transtibial pullout and suture anchor techniques. As a result of the meniscal root healing status, the suture anchor technique has a complete healing rate of about 85% and 65.7% for the transtibial pullout procedure. Several meniscal suture configurations have been proposed, each with different biomechanical stability. A technique using 2 simple sutures was shown to have lower root displacement, as well as greater stiffness, and was not statistically different from other suture techniques in a comparative study that included 2 simple sutures, horizontal mattress stitches, a modified Mason-Allen configuration, and 2 modified loop stitches.^14^^,^^18^ LaPrade et al.^19^ have described repair of the medial meniscal root using a double-tunnel pullout technique, and patients have reported improvements in pain, function, and activity level. To better restore the native anatomic footprint and improve biological regeneration, single- and double-tunnel techniques have been described.^18^

After the reduction stitch suture has been performed, the extrusion reduction is also reduced. This technique helps to reduce stress at the repair site, improve shock absorption efficiency, and reduce the development of osteoarthritis in the future. At short-term follow-up, patients were found to have improved Lysholm and International Knee Documentation Committee scores, as well as a decrease in Kellgren-Lawrence grading, evident from knee radiographic imaging.^20^ However, the issue of meniscal extrusion correction after root repair remains uncertain and debatable, requiring a separate treatment to address it.

MMRs are necessarily performed in well-selected patients. The triple-loaded soft anchor repair technique offers a strong suture construction; less displacement; no need to access the posterior compartment of the knee, which contains the neurovascular bundle; and improved contact surface healing. Moreover, it is a familiar arthroscopic technique. For coexisting special conditions (anterior cruciate ligament or PCL reconstruction and HTO), we still recommend performing MMR with other auxiliary procedures because this technique eliminates tunnel jamming during the other procedures. All of these factors contribute to better clinical and radiologic outcomes.

## Supplementary Data

ICMJE author disclosure forms

Video 1 Surgical technique. In this patient, a completely torn posterior medial meniscal root was diagnosed based on the clinical presentation, as well as magnetic resonance imaging (T2-weighted fat-saturated coronal view) showing the presence of the cleft sign. The patient is positioned supine with the surgical leg in 90° of knee flexion. We used 3 portals and an anteromedial proximal tibial mini oblique incision. The standard anterolateral (AL) and anteromedial (AM) parapatellar portals directly adjacent to the patellar tendon are made together with accessory mid-medial portals. An 18-gauge needle is used to perform percutaneous medial collateral ligament (MCL) release. The landmark is above 1.2 cm from the intersection point of the posterior border of the proximal tibia line and parallel to the joint line. The knee is flexed to 20° while a valgus force is applied to the tibiofemoral joint and external rotation is applied to the foot. The medial working space and visualization are significantly improved. The insertion guidance mid-medial portal uses the transillumination point from the arthroscopic light source at the upper part of the medial meniscal body. By use of a ring curette via the mid-medial portal, the footprint of the medial meniscal root is debrided, and the cartilage is removed until the subchondral bone is exposed. A low-profile aiming device (Acufex Director Drill Guide) uses the guidance from the AM proximal tibia to the posteromedial meniscal root footprint. The tibial tunnel drill hole was made by using a 2.9 mm cannulated drill. A No. 2-0 polydioxanone loop (No. 2-0 PDS II suture) is placed into the cannulated drill at the AM proximal tibia to the intra-articular of the knee. The suture retriever is used to retrieve the No. 2-0 PDS to the outside via the AM portal. The suture tape with a triple loaded suture anchor is pulled into the meniscal root footprint until it reaches the 2 cm depth of the soft suture anchor. The tip of a triple-loaded soft anchor (Y-Knot PRO RC, 2.8 mm) is sutured with fiber tape (Ultratape). The soft anchor is deployment by the surgeon pulled the suture limbs and soft anchor suture becomes from the spindle shape to the ball shape with secure fixation into the tibia tunnel. The FirstPass Mini Suture Passer performs suturing at the 3-mm medial part of the medial meniscal root tear site. The second horizontal stitch is sutured far from the first stitch, by approximately 5 mm. The FirstPass Mini Suture Passer is loaded with a looped suture limb, allowing a loop to be passed up through the posteromedial part of the meniscal root. The lateral compression stitch is performed repeatedly step by step with the FirstPass Mini Suture Passer. This suture limb undergoes retrograde suturing far from the first horizontal stitch, around 5 mm. The knot-tying procedure should be performed in the following sequence: The first knot tied is the reduction stitch knot, the second is the medial compression stitch knot, and the last is the lateral compression stitch knot. The first reduction stitch is tied by a knot pusher, and the post of the knot pusher is set at the medial suture limb of the reduction stitch. Knot tying should be performed medial, close to the intercondylar notch, to prevent damage to the medial femoral cartilage by the knot. The second knot tying involves the medial compression stitch, for which the post of the knot pusher should be in the posterior part of the medial meniscal root. The last knot tying involves the lateral compression stitch, with the previous steps repeated one by one.
